# When too late is too late: timing of CAR T-cell therapy in SLE & lupus nephritis

**DOI:** 10.3389/fimmu.2026.1914091

**Published:** 2026-09-24

**Authors:** Saira Sheikh, Shaun Jackson, Vimal Derebail, Natalie Grover, Astia Allenzara

**Affiliations:** 1 School of Medicine, Department of Medicine, Division of Rheumatology, Allergy and Immunology & University of North Carolina (UNC) Thurston Arthritis Research Center, University of North Carolina at Chapel Hill, Chapel Hill, NC, United States; 2 Department of Pediatrics, University of Washington School of Medicine, Seattle, WA, United States; 3 Seattle Children’s Research Institute, Seattle, WA, United States; 4 School of Medicine, Department of Medicine, Division of Nephrology and Hypertension and University of North Carolina (UNC) Kidney Center, University of North Carolina at Chapel Hill, Chapel Hill, NC, United States; 5 School of Medicine, Department of Medicine, Division of Hematology, University of North Carolina at Chapel Hill, Chapel Hill, NC, United States

**Keywords:** cell therapy, chimeric antigen receptor (CAR T), clinical trials, lupus (SLE), lupus nephritis, treatment

## Abstract

CAR T-cell therapy has demonstrated the capacity to induce deep, drug-free remissions in patients with severe, refractory systemic lupus erythematosus (SLE), including those with lupus nephritis (LN). These observations challenge long-standing assumptions about the reversibility of autoimmune disease and raise a critical question: whether CAR T-cell therapy should remain a therapy of last resort or if trials could investigate use earlier in the disease course, when its disease-modifying potential may be greatest. When CAR T-cell therapy is administered late in the disease course, its true biologic effect may therefore be underestimated, and clinical nonresponse may be misattributed to lack of therapeutic activity rather than to persistent damage and fibrosis. CAR T-cell therapy is still experimental and undergoing investigation as a treatment option in autoimmune diseases. Given the potential for a durable disease-free, drug free remission, we propose investigating use of CAR T-cell therapy in early severe SLE. We highlight CAR T-cell therapy in SLE and LN with a brief discussion of risks and benefits from the evidence to date. The current positioning as a refractory/salvage therapy late in the treatment algorithm may limit its impact for patients with SLE at high risk of irreversible organ damage.

Chimeric antigen receptor (CAR) T-cell therapy has recently demonstrated the capacity to induce deep, drug-free remissions in patients with severe, refractory systemic lupus erythematosus (SLE/lupus) including those with lupus nephritis (LN). These observations challenge long-standing assumptions about the reversibility of autoimmunity and raise a critical question: whether CAR T-cell therapy should remain a therapy of last resort, or if treatment should be offered earlier in the disease course when disease-modifying potential may be greatest. Due to the potential for unpredictable long-term effects, the Food and Drug Administration (FDA) has generally advised CAR T-cell therapy to be used in refractory disease defined by failure of two non-glucocorticoid, non-hydroxychloroquine immunosuppressants (1). However, the typical SLE patient enrolled in an investigational CAR T-cell trial has disease that is both more refractory and long-standing than required by regulatory bodies or study protocols with a median of 5.5 therapies (interquartile range 5.0-8.2) (2). Thus, CAR T-cell therapy is currently being offered even later in the disease course than mandated by trial protocols, after patients have cycled through and exhausted numerous medications. With such length of time of ongoing disease activity, patients are more likely to have greater cumulative end-organ damage, that would be less-reversible.

Current SLE and LN clinical guidelines recommend the chronic use of multiple concurrent immunosuppressive therapies (3). Despite recent FDA approval of new therapies for LN, less than half of patients with LN are able to achieve long term remission (4). Although these approaches can control inflammatory activity, many patients experience relapsing disease, cumulative treatment toxicity, and progressive organ damage. Importantly, these therapies suppress immune activity without fundamentally reprogramming the autoreactive immune repertoire.

In contrast, CD19-directed CAR T-cell therapy offers a mechanistically distinct approach by eliminating autoreactive B-cell populations and enabling immune reconstitution, with the potential for durable restoration of immune tolerance (5, 6). The biological basis underlying the transformative efficacy of CD19 CAR T-cell therapy relative to conventional anti-CD20 monoclonal antibodies has yet to be fully elucidated. However, two complementary explanations have been proposed. First, even Type II anti-CD20 agents optimized for superior B cell depletion, are likely ineffective at completely depleting B cells within secondary lymphoid organs (7). Second, CD19 targeting may confer additional benefit over anti-CD20 monoclonal antibodies by eliminating plasma cells that retain CD19 expression but lack CD20, a population that is not limited to actively dividing plasmablasts (6). Additionally, BCMA-directed approaches may offer complementary or alternative mechanisms for eliminating long-lived plasma cells that are resistant to CD19- and CD20-targeted approaches and are being investigated in this evolving landscape of B-cell depletion strategies.

There is strong biological rationale for earlier intervention with CAR T-cell therapy. B cells play a central role in the early pathogenesis of SLE through autoantibody production, antigen presentation, and amplification of pathogenic T-cell responses. In early disease, immune dysregulation predominates; in later stages, disease manifestations increasingly reflect irreversible structural injury. In LN, chronic glomerulosclerosis, interstitial fibrosis, and tubular atrophy represent permanent damage that is unlikely to improve even in the setting of complete immunologic remission. As a consequence, the apparent clinical efficacy of CAR T-cell therapy may be reduced in patients with extensive preexisting organ damage. Persistent proteinuria, impaired kidney function, or ongoing symptoms after therapy may not indicate failure of immune reprogramming but rather the presence of irreversible parenchymal injury. When CAR T-cell therapy is administered late in the disease course, its true biologic effect may therefore be underestimated, and clinical nonresponse may be misattributed to lack of therapeutic activity rather than to persistent damage and fibrosis.

The rapidly evolving landscape of CAR T-cell therapy raises the possibility that its role in SLE and LN may ultimately extend beyond a last resort therapy for highly refractory disease. The greatest potential benefit may lie in carefully selected patients with moderate-severe, organ-threatening disease who retain substantial salvageable organ function, where immune reset could prevent irreversible damage rather than merely treat its consequences. Prospective studies are needed to determine whether intervening earlier in the disease course improves long-term renal and systemic outcomes compared with continued sequential immunosuppression.

In our hands (8), similar to other groups, we have seen remarkably favorable outcomes of CD19 CAR T-cell therapy in patients with severe SLE and LN (9). An exception to this experience has been patients who have long-standing LN with evidence of pre-existing damage on kidney biopsy. An illustrative case from our group is a young man with treatment-refractory class IV LN who exhibited significant proteinuria (urine protein: creatinine ratio (uPCR); 6.9 mg/mg) and reduced kidney function (estimated glomerular filtration rate (eGFR); 30ml/min/1.73m (2)) despite treatment with prednisone, mycophenolate, hydroxychloroquine, and cyclophosphamide. A baseline renal biopsy obtained prior to CD19 CAR T-cell therapy showed both high disease activity (modified NIH activity index 17/24) and significant pre-existing kidney damage (chronicity index 6/12). Importantly, this subject did not carry APOL1 G1 or G2 risk alleles associated with chronic kidney disease (CKD) progression (10).

The patient was enrolled into a clinical trial evaluating autologous CD19 CAR T-cell therapy. At screening visit he had a Systemic Lupus Erythematosus Disease Activity Index (SLEDAI) of 12 driven by proteinuria, hematuria, pyuria and dsDNA antibodies. He received lymphodepleting chemotherapy which consisted of fludarabine 18 mg/m (2)and cyclophosphamide 300 mg/m (2) administered for 3 consecutive days 72 hours before CAR T-cell infusion. The patient tolerated the lymphodepleting therapy and CAR T- cell infusion without Cytokine Release Syndrome (CRS), ICANS (Immune Effector Cell-Associated Neurotoxicity Syndrome), infections, hematologic toxicity, or other significant adverse events. The patient had adequate CAR T-cell expansion and evidence of peripheral B cell depletion. Following CAR T-cell treatment, he endorsed a significant improvement in fatigue and quality of life. However, he had persistent proteinuria and positive dsDNA with a sustained SLEDAI score of 6. Approximately six months after CAR T-cell infusion, he had a repeat renal biopsy due to persistent proteinuria and an elevated serum creatinine. Kidney histopathology was notable for persistent active LN, characterized by endocapillary hypercellularity, focal karyorrhexis, and focal fibrinoid necrosis in most glomeruli, as well as 10% cellular and 40% fibrocellular crescents. Compared to the biopsy obtained 6 months prior to CAR T-cell therapy, the kidney exhibited advanced chronicity including severe interstitial fibrosis and tubular atrophy, diffuse glomerular sclerosing features involving 80% of the glomeruli, and 30% fibrous crescents. Histopathologic scoring showed a modified NIH activity index of 12/24 and chronicity index of 11/12. The patient subsequently progressed to end-stage kidney disease requiring hemodialysis.

Given the potential for LN to progress rapidly and unpredictably from active inflammation to irreversible chronic kidney damage, early identification and treatment is critical. In patients with moderate to severe, organ-threatening disease, particularly those with substantial active inflammation but limited irreversible renal damage, early consideration of CAR T-cell therapy may offer an opportunity to achieve durable immune disease control before cumulative organ damage becomes irreversible. Conversely, in patients with well-controlled disease or predominantly established chronic renal scarring, the benefit of earlier CAR T-cell therapy remains uncertain and must be weighed against the risks of cellular therapy.

Activity and chronicity should be viewed as overlapping dimensions rather than binary states in LN. Kidney biopsy can demonstrate potentially reversible active lesions, including endocapillary hypercellularity, necrosis, cellular/fibrocellular crescents, and interstitial inflammation alongside chronic lesions such as glomerulosclerosis, fibrous crescents, interstitial fibrosis, and tubular atrophy. The NIH activity index (scored from 0 to 24) and chronicity index (0 to 12) provide a standardized framework for characterizing this balance and can complement clinical measures of disease activity and renal function (11). Increasing chronicity, particularly extensive interstitial fibrosis and tubular atrophy, is associated with diminished potential for renal recovery. Recent consensus work has identified activity index ≥4 and chronicity index ≥4 as markers associated with worse renal outcomes, although these should not be regarded as absolute thresholds of treatment futility (12). Accordingly, the rationale for earlier intervention is not predicated on a strict separation between activity and chronicity. Rather, it is based on recognizing a therapeutic window in which substantial active inflammation and risk of progression remain present, while sufficient viable renal parenchyma remains to permit meaningful recovery. For patients with moderate-to-severe LN at high risk of progression, intervening earlier offers a compelling opportunity to preserve renal function rather than waiting until irreversible damage accumulates and predominates. The ultimate objective is to explore a paradigm shift in treatment earlier in the trajectory, before cumulative chronic injury substantially limits the capacity for recovery.

Patients face numerous obstacles to care including access to subspecialists and timely initiation of medications. Adhering to chronic immunosuppression can be challenging due to concerns about side effects, cost of medications and other logistical challenges. The potential to achieve a drug-free and disease-free remission after a single infusion of CAR T-cells is an exciting prospect for the field. We recognize that evaluating the efficacy of CAR T-cell therapies is complex and requires specialized expertise with investigator, industry and institutional investment, multidisciplinary collaboration, continuous patient engagement and long-term monitoring.

Earlier CAR T-cell therapy is unlikely to offer a clear advantage for all patients with SLE and LN. Patients with lower-risk disease who achieve a prompt and sustained response to standard therapy may have little incremental benefit from cellular therapy, and conventional treatment remains appropriate for many such patients. Our rationale for earlier CAR T-cell therapy is instead focused on patients with moderate-to-severe disease who are at high risk for progression, have inadequate or transient responses to standard therapy, or face substantial cumulative toxicity from ongoing immunosuppression. Practical barriers must also be considered, including the substantial cost of CAR T-cell therapy, manufacturing time, the need for specialized centers, and monitoring for complications such as CRS, ICANS, cytopenias, and infection. Access may be further limited by geographic, financial, transportation, and healthcare infrastructure barriers, particularly globally. Thus, earlier CAR T-cell therapy should be viewed as a risk-adapted strategy rather than a universal replacement for conventional therapy, with prospective studies needed to define the patients in whom its potential for durable disease control justifies its risks, costs, and logistical complexity.

It is important to acknowledge that limited access to specialized cellular therapy centers, transportation, time away from employment or caregiving responsibilities, and geographic and socioeconomic disparities could substantially restrict access, Manufacturing delays may also be clinically relevant in a disease in which renal injury can progress during the interval between treatment decision and administration. Future development of novel mechanistic and manufacturing approaches, alongside more flexible treatment models such as outpatient administration, may help reduce some of these barriers. Lessons from hematology/oncology, include nurse navigation, peer support, fertility counseling, transportation assistance, and lodging programs which could inform strategies to improve equitable access to cellular therapy.

The central question is whether the potential for durable disease modification justifies the risks, costs, logistical complexity, and opportunity costs of CAR T-cell therapy in a given patient. Prospective studies incorporating clinical response, histologic activity/chronicity, risk of disease progression, patient preferences, cost-effectiveness, and access will be essential to define the appropriate population and timing.

At this time, CAR T-cell therapy is still experimental and undergoing investigation as part of clinical trials for autoimmune diseases. Given the potential for durable disease-free, drug free remission, we propose incorporating clinical trial discussion as part of routine high-quality SLE care (13) and specifically considering CAR T-cell therapy in early, moderate to severe disease.

Experience across immune-mediated diseases supports the principle that earlier use of highly effective therapies improves long-term outcomes by preventing irreversible injury. Delaying CAR T-cell therapy until disease is refractory and organ damage is advanced may limit its measurable benefit and distort assessments of efficacy. This concern is particularly relevant as CAR T-cell therapy is being evaluated in small studies and early-phase trials, where patient selection strongly influences perceived outcomes.

CAR T-cell therapy and the lymphodepletion regimen carry both short-term and long-term risks. Infection risk is dependent on several factors, including the type of CAR T-cell product and target (e.g., BCMA versus CD19 targeting) and the use and type of lymphodepletion. Short term risks include CRS, ICANS, local immune effector cell-associated toxicity syndrome (LICATs), cytopenias and infection. Longer-term risks include persistent cytopenias, infections, and secondary malignancies. Most data for secondary malignancy after CAR T-cell therapy come from patients with hematologic malignancies due to longer follow-up time available. However, this population may not be reflective of patients with autoimmune disease because of differences in prior treatment exposures and potential baseline bone marrow abnormalities. In a systematic review of 5,517 patients with lymphoma or myeloma treated with CAR-T cell therapy, the median follow up was 21.7 months, and 5.8% of patients (326 total, 95% confidence interval 4.7-7.2) developed a secondary malignancy. Among these cases, 1.5% (5 total) were diagnosed with T-cell lymphomas, and only one of the five had the presence of the CAR transgene (14). The long-term risk profile in autoimmune disease remains uncertain because of limited duration of follow up data and lack of disease-specific data, particularly regarding secondary malignancies. These potential risks must be balanced against the cumulative harms of prolonged immunosuppression, including infection, malignancy, and progressive organ failure.

We hypothesize that earlier use of CAR T-cell therapy would allow treatment in patients with severe aggressive disease and greater physiologic reserve, fewer comorbidities, and a higher likelihood of demonstrable clinical benefit. In large B-cell lymphoma, CD19 CAR T-cell therapy is supported as a second line therapy (15), with clinical trials now investigating this therapy in the first line setting for high risk patients (16).

The possibility of a drug-free, disease-free remission provide a scientific rationale for prospective trials evaluating CAR T-cell therapy earlier in the disease course for patients with severe SLE and LN who are at high risk for progression and end-organ damage. Future clinical trials should evaluate CAR T-cell therapy not as a salvage treatment but rather as an option earlier in the disease course. This approach could employ stratification by risk of disease progression and degree of preexisting organ damage, and distinguish immunologic remission from recovery of organ function. Without such an approach, the field risks conflating irreversible disease damage with reduced apparent efficacy, potentially delaying adoption of a therapy capable of fundamentally altering the natural history of SLE and LN.

## Data Availability

The original contributions presented in the study are included in the article/supplementary material. Further inquiries can be directed to the corresponding author.
